# Functional dynamic modulation of colorectal cancer initiation and metastatic capacity by a novel MiR-7974 regulatory axis

**DOI:** 10.1016/j.bj.2025.100934

**Published:** 2025-11-28

**Authors:** Yu-Hao Liu, Yi-Tung Chen, Yu-Chang Chen, En Chin, Ying-Yu Lai, Chung-Pei Ma, Ian Yi-Feng Chang, Bertrand Chin-Ming Tan, Pei-Ting Hsu, Yi-Hsuan Lai, Wen-Sy Tsai, Chia-Yu Yang, Jau-Song Yu, Hung-Chih Hsu, Hsuan Liu

**Affiliations:** a Department of Cell and Molecular Biology, College of Medicine, Chang Gung University, Taoyuan, Taiwan; b Molecular Medicine Research Center, Chang Gung University, Taoyuan, Taiwan; c Department of Biomedical Sciences, College of Medicine, Chang Gung University, Taoyuan, Taiwan; d Graduate Institute of Biomedical Sciences, College of Medicine, Chang Gung University, Taoyuan, Taiwan; e Department of Neurosurgery, Lin-Kou Medical Center, Chang Gung Memorial Hospital, Taoyuan, Taiwan; f Research Center for Emerging Viral Infections, Chang Gung University, Taoyuan, Taiwan; g Division of Colon and Rectal Surgery, Lin-Kou Medical Center, Chang Gung Memorial Hospital, Taoyuan, Taiwan; h Department of Microbiology and Immunology, College of Medicine, Chang Gung University, Taoyuan, Taiwan; i Department of Otolaryngology-Head & Neck Surgery, Chang Gung Memorial Hospital, Linkou, Taoyuan, Taiwan; j Liver Research Center, Chang Gung Memorial Hospital at Linkou, Taoyuan, Taiwan; k Division of Hematology-Oncology, Department of Internal Medicine, Chang Gung Memorial Hospital at Linkou, Taoyuan, Taiwan; l College of Medicine, Chang Gung University, Taoyuan, Taiwan

**Keywords:** Colorectal cancer, MiR-7974, Metastatic potential, Cancer development

## Abstract

**Background:**

Colorectal cancer (CRC) is one of the most prevalently diagnosed malignancies. Frequent metastasis and recurrence render treatments ineffective. The accumulation of omics data has helped develop a comprehensive functional regulatory network underlying tumorigenesis, causing significant breakthroughs in cancer therapy.

**Methods:**

Systematic transcriptomic analysis of CRC tissues and matched normal samples identified miR-7974 as a novel miRNA with distinct expression pattern in CRC. Independent RT-qPCR assays further validated its tumor-biased expression. To investigate the role of miR-7974 in tumor progression, a series of cell-based assays and xenograft models were conducted. Additionally, RNA sequencing, reporter assays, and functional rescue experiments were performed to delineate the downstream regulatory network, with specific focuses on the miR-7974/CDKN1A and miR-7974/MYO1E axes involved in tumor growth and metastasis.

**Results:**

MiR-7974 demonstrated high expression in early CRC but decreased abundance in later stages. We uncover that miR-7974 augments cancer growth by mitigating the expression of the cell cycle regulator, CDKN1A, through in vitro assays. Concurrently, miR-7974 reduces cellular migration and invasion by targeting MYO1E. In-depth transcriptomic investigations revealed miR-7974's role in repressing the epithelial-to-mesenchymal transition (EMT) in CRC cells, thereby maintaining the tumor in a highly proliferative epithelial state. This result is congruent with miR-7974's pronounced expression in early-stage CRC and its attenuation in advanced, metastatic stages. Such dynamic changes in expression patterns elucidate miR-7974's prognostic significance. Despite its abundant expression in CRC tissues, patients with heightened miR-7974 levels achieved more favorable survival outcomes.

**Conclusions:**

Our findings demonstrate that miR-7974 regulates EMT plasticity, promoting a rapidly proliferating epithelial phenotype while reducing metastatic potential in CRC. Dynamic miR-7974 expression, coupled with its associated target gene regulation, provides the mechanistic foundation for understanding the acquisition of metastatic potential. This emphasizes the functional effect of miR-7974 on CRC growth and provides a deeper understanding of miRNome dynamics during cancer development.

## Highlights

• Transcriptomic profiling discovers the tumor-associated miR-7974 expression.
• MiR-7974 targeting to CDKN1A expression promotes colorectal cancer growth.
• MYO1E Suppression by miR-7974 regulates cancer cell metastatic potential.
• Dynamic miR-7974 expression favors colorectal cancer metastasis and progression.

## Graphical abstract

**Figure:**
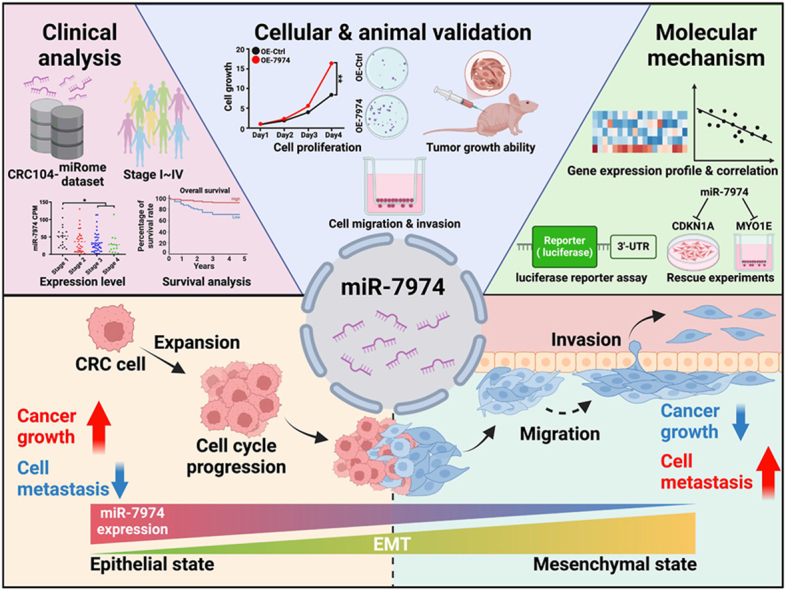

## 1 Introduction

Colorectal cancer (CRC) is one of the most devastating cancers globally, causing hundreds of thousands of deaths annually [1]. CRC remains the third leading cause of cancer death globally despite surgical resection and advances in radiotherapy and chemotherapy [2]. In Taiwan, CRC is the most prevalent cancer in males and the second most predominant in females. CRC initiation is a multifaceted biological process that involves a series of genomic and epigenomic alterations occurring over an extended period. Early detection of CRC, during its potentially curable stages, can significantly decrease the mortality rate associated with the disease [3]. Although considerable progress has been made in uncovering the molecular mechanisms and available treatment options for CRC, overall survival rate for CRC patients have shown limited improvement in recent years. Further research into novel oncogenic signaling pathways is urgently needed to identify new therapeutic targets, as many patients with advanced CRC do not respond effectively to current treatment regimens. The complete functional genomic regulatory network underlying tumorigenesis has been progressively constructed with the growing cancer omics data, causing many breakthroughs in cancer treatment [[4], [5], [6]].

In recent years, cumulative research has revealed noncoding RNA constituents, particularly microRNAs [[7], [8], [9]], acting as key regulators in CRC development and malignancy. Hence, these constituents further serve as reporters for CRC progression [10,11]. MicroRNAs (miRNAs) are categorized as small noncoding RNA molecules approximately 22 nucleotides in length that are transcribed by RNA polymerase II and undergo RNase processing to form mature miRNAs [12]. MiRNAs bind to the 3’ untranslated region (UTR) of target genes, through their seed sequences, to mediate target mRNA degradation in a post-transcriptional manner [13]. Recently, miRNAs have been implicated as oncogenes and suppressors to fine-tune cancer progression, especially in CRC. In particular, miR-26b targets PTEN expression, facilitating CRC metastasis and stemness capacity [14]. Overexpression of miR-10b promotes CRC malignancy that downregulates E-cadherin expression and promotes TWIST expression [15]. Mounting evidence supports the association of deregulated miRNA expression and CRC tumor progression and distant metastasis [16]. These findings indicate a comprehensive miRNome profiling and thorough clarification of their molecular properties may provide better insight into this public health concern.

Epithelial-to-mesenchymal transition (EMT) is a reversible process in which cells lose their epithelial properties and acquire mesenchymal characteristic, such as increased motility. The transition has been functionally linked to the metastatic phenotype of malignant carcinomas [17,18]. Several signaling pathways in CRC have been implicated in promoting EMT. Transforming growth factor-beta signaling, known for its role in tumor progression, induces the expression of EMT markers such as SNAIL and Vimentin. In parallel, the WNT/β-catenin pathway, when aberrantly activated, contributes to EMT-associated de-differentiation, particularly at the invasive front of CRC tumors. Additionally, HIF-1α has been shown to facilitate EMT and enhance cell invasiveness by transcriptionally activating ZEB1, a key EMT-inducing transcription factor. Furthermore, recent studies suggest that extracellular vesicles may play a key role in facilitating tumor state reversion, thereby aiding metastasis [9,19,20]. Additionally, extracellular matrix citrullination mediated by small extracellular vesicles has been implicated with hepatocellular metastasis [19]. Numerous studies recognize the indicative role of exosomal miR-200 family in tumor prognosis and metastasis, demonstrating the significance of the regulated mesenchymal-to-epithelial transition (MET) signaling by exosomal miRNome [[21], [22], [23]]. Notably, miR-200 levels are significantly upregulated in liver metastasis tissue compared to primary tumor tissue, where they target ZEB1 gene expression to induce MET transition in metastatic cells [24].

In our previous work, we cataloged distinctions in miRNA-mRNA interactions between tumors and matched normal tissues from 104 CRC patients [6]. From this dataset, we identified miR-7974 as a novel cancer-associated miRNA with stage-specific expression patterns in CRC. Unlike other miRNAs in CRC, which commonly drive both proliferation and metastasis, miR-7974 uniquely sustains cells in a highly proliferative epithelial state while limiting metastatic potential. This function fills an important gap in CRC research, as most EMT-associated miRNAs promote metastasis, whereas miR-7974 appears to regulate early tumor growth without advancing metastasis. Our findings reveal that miR-7974 controls EMT plasticity by precisely modulating the EMT-MET transition in a spatiotemporal manner, maintaining an epithelial phenotype and reducing invasion in early-stage CRC. These results highlight miR-7974's unique regulatory role in CRC progression, positioning it as a promising prognostic and therapeutic target while providing insights into miRNome dynamics.

## 2 Materials and methods

### 2.1 Cell culture

HCT-116 (BCRC #60349), LoVo (BCRC #60148), SW480 (BCRC #60249), and SW620 (BCRC #60343) cell lines were purchased from Bioresource Collection and Research Center of the Food Industry Research and Development Institute (Hsinchu, Taiwan). We cultivated HCT116 cells in McCoy's 5A medium supplemented with 10 % fetal bovine serum (FBS), and cultured LoVo cells in F-12 basal medium containing 20 % FBS. HCT-116 and LoVo cell were predominantly used for cell-based experiments based on their genetic background. We maintained SW480 and SW620 cells in F-15 basal medium containing 10 % FBS and 2 μM L-glutamine. HCT116 and LoVo cells were incubated in 5 % CO_2_ at 37 °C, whereas SW480 and SW620 cells were maintained in 0 % CO_2_ at 37 °C. All media were purchased from Thermo Fisher Scientific, and FBS was heat-inactivated before adding to medium.

### 2.2 Construction for gene expression and knockdown

BLOCK-iT™ HiPerform™ Lentiviral Pol II miR RNAi Expression System with EmGFP (ThermoFisher Scientific) was adopted for miRNA overexpression in cells. The sequences of miR-7974 and miR-LacZ were synthesized and constructed into pLenti6.4 vectors, and the plasmids were subjected to lentiviral package and further cell infection based on the operation manual. For the knockdown of miR-7974, we synthesized the specific LNA-oligonucleotides to target miR-7974 expression, and the sequence-scrambled LNA was used for control transfection. The CDKN1A expression plasmid was purchased from Origene, and MYO1E gene was amplified from cDNA template and subcloned into expression vector. All used primer sequences were listed in [Supplemental Table S1].

### 2.3 RNA extraction, reverse transcription and quantitative PCR (RT-qPCR)

Total RNA was extracted by the TRIzol reagent (Thermo Fisher Scientific) following the manufacturer's protocol, and cDNA synthesis was performed via Superscript III reverse transcriptase with random hexamers or miRNA-specific primers. Gene expression in the cDNA samples was quantified using real-time quantitative PCR (iQ5 Gradient Real-Time SYBR-Green PCR system) with target-specific primers. The relative expression levels were evaluated by CFX Manager software (Bio-Rad, CA, USA), via the normalization to the expression of housekeeping genes, RNU6 and EEF1A1. The clinical samples used for RT-qPCR analysis were obtained under ethical approval (IRB No. 103-2529B). Cohort 1 (1^st^ cohort) consisted of samples that had previously undergone NGS-based transcriptome profiling (accession number: PRJNA387172), whereas cohort 2 (2^nd^ cohort) included additional samples that were not subjected to NGS analysis. Primer specificity was analyzed and assessed by gel electrophoresis, and the designed primer sequences were showed in the [Supplemental Table S1].

### 2.4 Cell proliferation analyses and colony formation assays

For the cell proliferation analysis, MTT reagent (Sigma-Aldrich) was used to assess cell viability. Briefly, cells were seeded in 96-well plates and incubated at the indicated time-points, and the MTT substrate was added to analyzed cells for 1h incubation. Next, culture medium was discarded, and DMSO was added to dissolve formed purple crystal. Subsequently, the product was measured by spectrometer at the wavelength of 570 nm. The obtained values were normalized to the value of Day1 for depicting growth curve. In colony formation assay, cells were seeded into 6-well plates with the indicated cell numbers (HCT116: 100; LoVo: 1000), and the seeded cells were incubated within 12–14 days for colony formation. Formed colonies were fixed with methanol and stained by crystal violate solution, and the colonies were counted as the indication of colony formation ability.

### 2.5 Cell cycle analysis

Cells were harvested and fixed by 70 % ethanol for 4 h at −20 °C, and the fixed cells were permeabilized by reagent (0.5 % TritonX-100, 2 % RNase) and subsequently stained with PI reagent (50 μg/ml) for 30 min. Stained cells were subjected to flow cytometry (BD FACSCalibur) to analyze cell phase distribution. Each cell phase was normalized and showed in percentage bar graph.

### 2.6 Mouse xenograft models

Male BALB/cAnN.Cg-Foxn1nu/CrlNarl mice (8-week-old) provided by National Laboratory Animal Center were used for animal study. Specific cell lines were mixed with Matrigel and inoculated into flank of mice. Tumor formation and progression was assessed and measured by a Vernier caliper, and the formed tumor volume was calculated at high x width^2^ x 0.52. Mice were sacrificed at the indicated time-point, and the formed tumors were dissected and weighted.

### 2.7 Transwell migration and invasion assays, and wound healing assay

For Transwell assays, HCT116 (1 x 10^5^) and LoVo (3 x 10^5^) cells were seeded into Transwell polystyrene membrane insert chamber (Corning, NY, USA) coated with Matrigel (invasion) or without (for migration), and the lower level was filled with culture medium. After 20 h incubation, the migrating (invading) cells through chambers were fixed and subsequently stained by crystal violet for 30 min at room temperature. Migrating or invading cells were quantified by microscope. In wound healing assay, cells were collected and seeded into Culture-Insert 2 well (Blossombio) at 6-well plate. After 24 h incubation, Culture-Insert 2 well was removed, and the wound healing process was recorded by the Cytation™ 5 Cell Imaging instrument (BioTek). Migratory capacity was measured in the percentages of remaining wound area that was normalized to the start site.

### 2.8 Luciferase reporter assays

For reporter vector construction, the 3’ UTR regions of CDKN1A and MYO1E genes were amplified from cDNA templates and subcloned into pMIR-REPORT-luciferase vectors (pMIR-REPORT™ system, Applied Biosystems), and mutant constructs were generated by site-directed mutagenesis. Molecular cloning primer sequences were listed in [Supplemental Table S1]. Reporter constructs were transfected into cells, and the cells were harvested and subjected to Dual-Luciferase® Reporter Assay System (Promega) for the evaluation of the miRNA targeting efficiency.

### 2.9 RNA sequencing and data analysis

Before sequencing, the quality of the extracted RNA was evaluated using the RNA Nano 6000 Assay Kit on the Agilent platform (Agilent Technologies, CA, USA). Sequencing libraries were prepared with the TruSeq RNA library preparation kit (Illumina), following the manufacturer's protocol. The concentration and size distribution of the libraries were assessed using the Agilent 2100 Bioanalyzer with the High Sensitivity DNA Assay (Agilent Technologies). Equal amounts of each library were then analyzed using the NextSeq 500 sequencer (Illumina). Raw sequencing data were processed through BaseSpace Sequence Hub (Illumina) for adaptor trimming, after which the data were aligned to the *Homo sapiens* genome (hg38 version). The resulting BAM files were imported into Partek Flow software for read quantification and differential gene expression analysis. Genes showing at least a 1.5-fold change (|FC| ≥ 1.5) and a P-value <0.1 were filtered, identifying 285 upregulated and 488 downregulated genes. Hierarchical clustering and pathway enrichment analysis of these genes were performed using Ingenuity Pathway Analysis (QIAGEN), and miRNA target prediction was conducted using TargetScan.

### 2.10 Establishment of the highly progressively HCT116 cell line

To further interpret gene remodeling in CRC progression, we employed the Transwell system to select progressively more migrating cancer cells based on the reference article [25]. Through four-round collecting the migrated cells across the matrigel-coated Transwell membranes, we established the higher migration HCT116 M4 cells that was designated by the four-round selection. cells. The parental HCT116 cells was accordingly termed as M0 cells.

### 2.11 Statistical analysis

The Kaplan–Meier survival analysis was employed to evaluate the relationship between markers and overall survival, with statistical comparisons conducted using the Wilcoxon test. Pearson's correlation methodology was employed to evaluate the correlation between the expression of target miR-7974 and EMT scores [26]. Statistical significance was expressed as P-values.

### 2.12 Retrieval and analysis of public databases

Small RNA and mRNA sequencing data from CRC tissues were obtained from our previously published study and are publicly available in the NCBI Sequence Read Archive (SRA) under accession number PRJNA387172 [6]. This dataset was utilized to identify differentially expressed miRNAs and mRNAs in CRC specimens. Additionally, RNA sequencing data from The Cancer Genome Atlas (TCGA) were downloaded via the Genomic Data Commons (GDC) Data Portal. Data corresponding to colon adenocarcinoma (COAD) and rectal adenocarcinoma (READ) cases were retrieved, along with matched clinical and recurrence information. TCGA datasets were processed using standardized pipelines. Overall survival was defined as the interval from the date of initial diagnosis to the date of death or last follow-up.

### 2.13 Ethics approval and consent to participate

This study was approved by the Chang Gung Memorial Hospital Institutional Review Board as a retrospective analysis (IRB 103-2529B) and was conducted within the guidelines of the Declaration of Helsinki. Patients/families were counseled with regard to the present study design, and all participants provided written informed consent to participate in the study.

## 3 Results

### 3.1 Identification of tumor-associated miR-7974 expression from CRC miRNome profiling

**Fig. 1:**
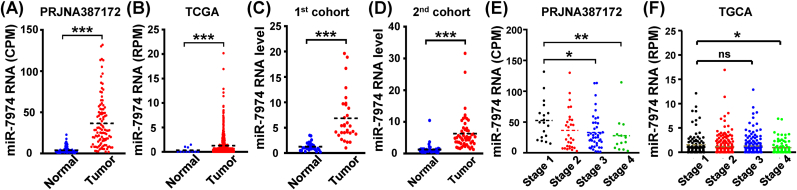
**MiRNA transcriptome landscape in colorectal cancer tissues.** (A) The read counts of miR-7974 transcript in normal and tumor sections of CRC-sequencing were depicted in the dot graph. (B) The reads of miR-7974 RNA in normal and tumor sections from TCGA database were shown in the dot graph. (C and D) RT-qPCR analyses of miR-7974 expression in the cohort samples were conducted and displayed in graphs. (E) The miR-7974 expression in the indicated tumor stage samples of CRC-sequencing were presented. (F) The miR-7974 expression in the indicated tumor stage samples from TCGA database were illustrated.

In our previous study, we systematically profiled the miRNA–mRNA regulatory network by comparing tumor tissues with matched adjacent normal samples from 104 CRC patients [6]. Heatmap analysis revealed distinct patterns of differentially expressed miRNAs [Fig. S1A], while pathway enrichment analysis indicated that these miRNAs were significantly associated with key oncogenic pathways, particularly Cell Cycle regulation and MicroRNAs in Cancer [Fig. S1B]. Among the dysregulated miRNAs, miR-7974 emerged as a particularly compelling candidate due to its substantial tumor-specific upregulation and limited existing characterization in the literature, highlighting its potential novel regulatory role in CRC. Subsequent analyses of miR-7974 expression levels confirmed its significant overexpression in CRC tumor tissues relative to normal counterparts in both our own RNA-seq dataset (PRJNA387172) [Fig. 1A] and the publicly available TCGA dataset [Fig. 1B]. To further validate the initial findings from our large-scale small RNA-seq experiments [6], we conducted independent RT-qPCR analyses using randomly selected 30 matched sample pairs from previously sequenced samples (1^st^ cohort) and additionally recruited 53 new matched sample pairs (2^nd^ cohort) [Fig. 1C and 1D]. Both independent validation cohorts consistently confirmed significantly elevated miR-7974 expression in tumor tissues. Interestingly, the analysis of miR-7974 expression across different TNM stages revealed a stage-dependent expression pattern: miR-7974 was markedly upregulated in early-stage CRC tissues compared with adjacent normal tissues, but its expression progressively and significantly declined with advancing stage in both datasets [Fig. 1E and 1F]. This dynamic expression pattern suggests a potential dual role for miR-7974 in modulating both tumor growth and metastatic potential in a stage-dependent manner, prompting further investigation into its specific regulatory mechanisms during CRC progression.

### 3.2 MiR-7974 promotes cell proliferation and enhances cell cycle progression

**Fig. 2:**
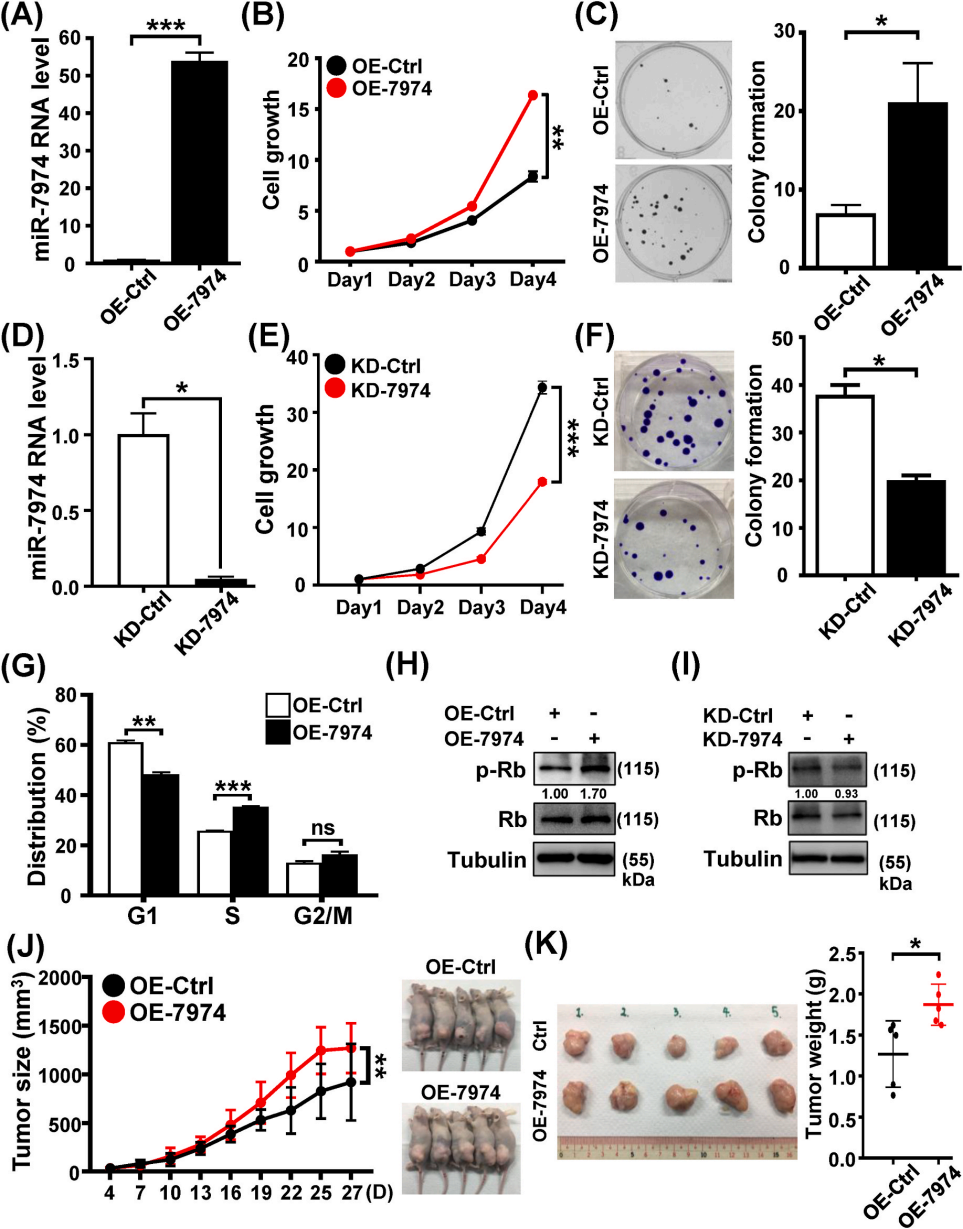
**MiR-7974 expression associates with colorectal cancer cell growth.** (A to C) MiR-7974 overexpression in HCT116 cells were conducted by infection, and the gene expression of cells was analyzed by RT-qPCR assay (A). Time-course cell proliferation (B) and colony formation ability (C) of the infected cells was measured by using MTT method and crystal violet staining, respectively. (D to F) MiR-7974 knockdown of cells were done and subjected to assays. The knockdown efficiency of cells was assessed by using RT-qPCR assays (D). Cell proliferation and clonogenity were assessed by MTT method and crystal violet staining, respectively. (G) Cell cycle analyses of the indicated cells were monitored by flow cytometry method. (H and I) The indicated protein expression of cells was analyzed by Western blot assays. (J) The mouse xenograft experiment was performed by inoculating specific cell lines, and tumors formed at the indicated time-points were dissected and recorded. Photographs of mice bearing tumors were shown. (K) The surgically removed tumor masses were shown, and tumor weight were depicted in a bar graph.

Based on its tumor-biased expression, we further investigated the functional role of miR-7974 in CRC progression. A lentiviral-based system was used to induce stable overexpression of miR-7974 (OE-7974) in HCT-116 cells, with miR-LacZ-infected cells serving as the negative control (OE-Ctrl). RT-qPCR analysis confirmed successful miR-7974 overexpression in HCT-116 cells [Fig. 2A], which exhibited significantly increased proliferation and colony-forming capacity as demonstrated by MTT and colony formation assays [Fig. 2B and 2C]. Consistent results were observed in another CRC cell line, LoVo, where miR-7974 overexpression similarly enhanced proliferative and clonogenic capacities [Fig. S2A and S2C]. To complement these findings, a specific LNA targeting miR-7974 (KD-7974) was synthesized and transfected into CRC cells to perform miR-7974 knockdown. RT-qPCR confirmed efficient suppression of miR-7974 expression [Fig. 2D]. Functional assays showed that miR-7974 knockdown reduced cell proliferation and colony-forming ability [Fig. 2E and 2F], reinforcing the growth-promoting role of miR-7974 in colorectal cancer cells.

To further dissect the mechanism of miR-7974-driven proliferation, we assessed its impact on cell cycle progression. Flow cytometry analysis showed that miR-7974 overexpression accelerated the G1–S phase transition in HCT-116 cells [Fig. 2G and S3], accompanied by elevated phosphorylation of the retinoblastoma protein (Rb), a key cell cycle checkpoint regulator [Fig. 2H]. Conversely, knockdown of miR-7974 reduced Rb phosphorylation [Fig. 2I]. Together, these results suggest that miR-7974 regulates cell cycle progression to promote CRC tumor growth.

To validate the pro-tumorigenic role of miR-7974 in vivo, we next employed a xenograft mouse model. The miR-7974 overexpressing and control cells were collected and subcutaneously injected into nude mice, and tumor formation was monitored at the indicated time points. Consistent with our in vitro findings, miR-7974 overexpression led to significantly enhanced tumor growth compared with the control group [Fig. 2J]. At the endpoint, tumors were harvested and measured, revealing a markedly higher average tumor weight in the miR-7974 group [Fig. 2K]. Taken together, our in vitro and in vivo findings provide compelling evidence that miR-7974 acts as an oncogenic regulator in CRC progression by promoting cell proliferation, clonogenic growth, and tumor expansion.

### 3.3 MiR-7974 exerts an inhibitory effect on colorectal cancer cell migration and invasion

**Fig. 3:**
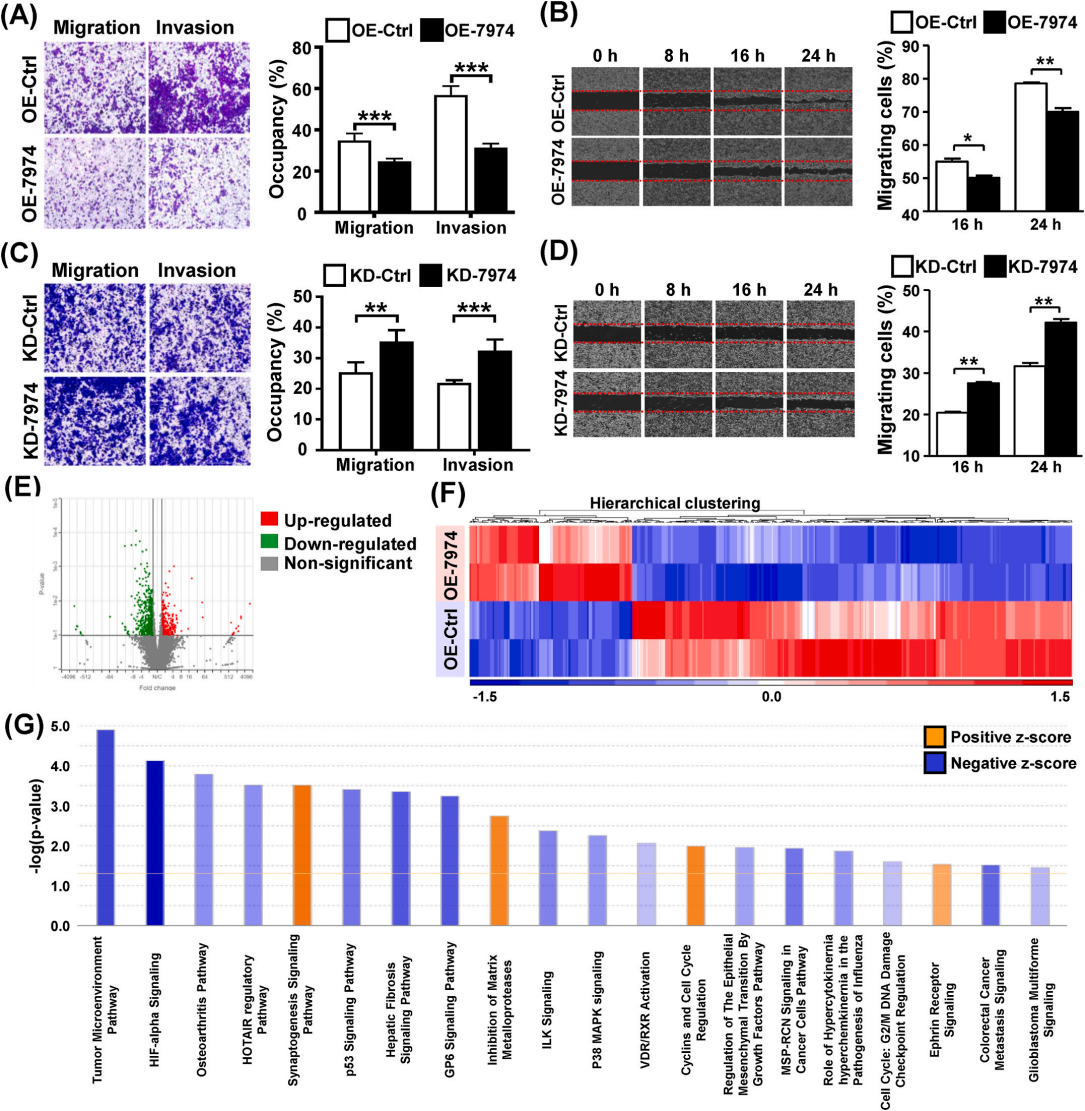
**MiR-7974 expression delivers negatively regulation on cell metastasis.** (A) MiR-7974 overexpression were done and subjected to Transwell migration and invasion analyses, and images and quantification of assays were shown. (B) Wound healing assays of indicated cells were performed by tip scratching, and the healing process was recorded with microscopy. (C and D) Cell metastasis of control and miR-7974 knockdown (KD-7974) cells were assessed by using Transwell assays and wound healing analyses. (E and F) RNA-sequencing assays of control and miR-7974 overexpressing cells were performed, and the differentially expressed genes were illustrated in volcano plot and heatmap representation. (G) Pathway annotations of the gene set by Ingenuity Pathway Analysis bioinformatics were showed.

To extend our investigation beyond proliferation, we next examined whether miR-7974 also influences metastatic properties in colorectal cancer cells. Transwell assays demonstrated that miR-7974 overexpression significantly impaired the migratory and invasive capacities of both HCT-116 and LoVo cells [Fig. 3A and S4]. This inhibitory effect was further supported by wound healing assays, in which overexpression of miR-7974 markedly delayed wound closure, indicating reduced cell motility [Fig. 3B]. Conversely, knockdown of miR-7974 using LNA-mediated inhibition enhanced HCT-116 cell motility, as evidenced by increased migration in both Transwell and wound healing assays [Fig. 3C and 3D]. These findings indicate that miR-7974 suppresses migratory and invasive phenotypes in CRC cells, in apparent contrast to its pro-proliferative role. These paradoxical observations suggest that miR-7974 may function in a stage- and context- dependent manner—being highly expressed in early-stage, epithelial-like CRCs characterized by enhanced proliferation, and downregulated in later-stage tumors with increased metastatic potential [Fig. 1E].

To further elucidate the molecular basis underlying the role of miR-7974 in CRC, we performed RNA sequencing analysis in HCT-116 cells stably overexpressing miR-7974 compared to control cells. Differentially expressed genes were identified (|fold change| ≥ 1.5, P-value < 0.1), resulting in 285 upregulated and 488 downregulated genes. The global expression changes were visualized using volcano plots and hierarchical heatmaps [Fig. 3E and 3F]. Pathway enrichment analysis using Ingenuity Pathway Analysis revealed significant activation of the cyclins and cell cycle regulation pathway, accompanied by suppression of signaling pathways associated with colorectal cancer metastasis and EMT [Fig. 3G and S5]. These transcriptomic findings are consistent with the phenotypic observations from our functional assays [Fig. 2, Fig. 3], further supporting a dual role for miR-7974 in promoting tumor growth through enhanced cell cycle progression while concurrently inhibiting metastatic capacity in colorectal cancer.

### 3.4 MiR-7974 modulates cell cycle by directly targeting CDKN1A

**Fig. 4:**
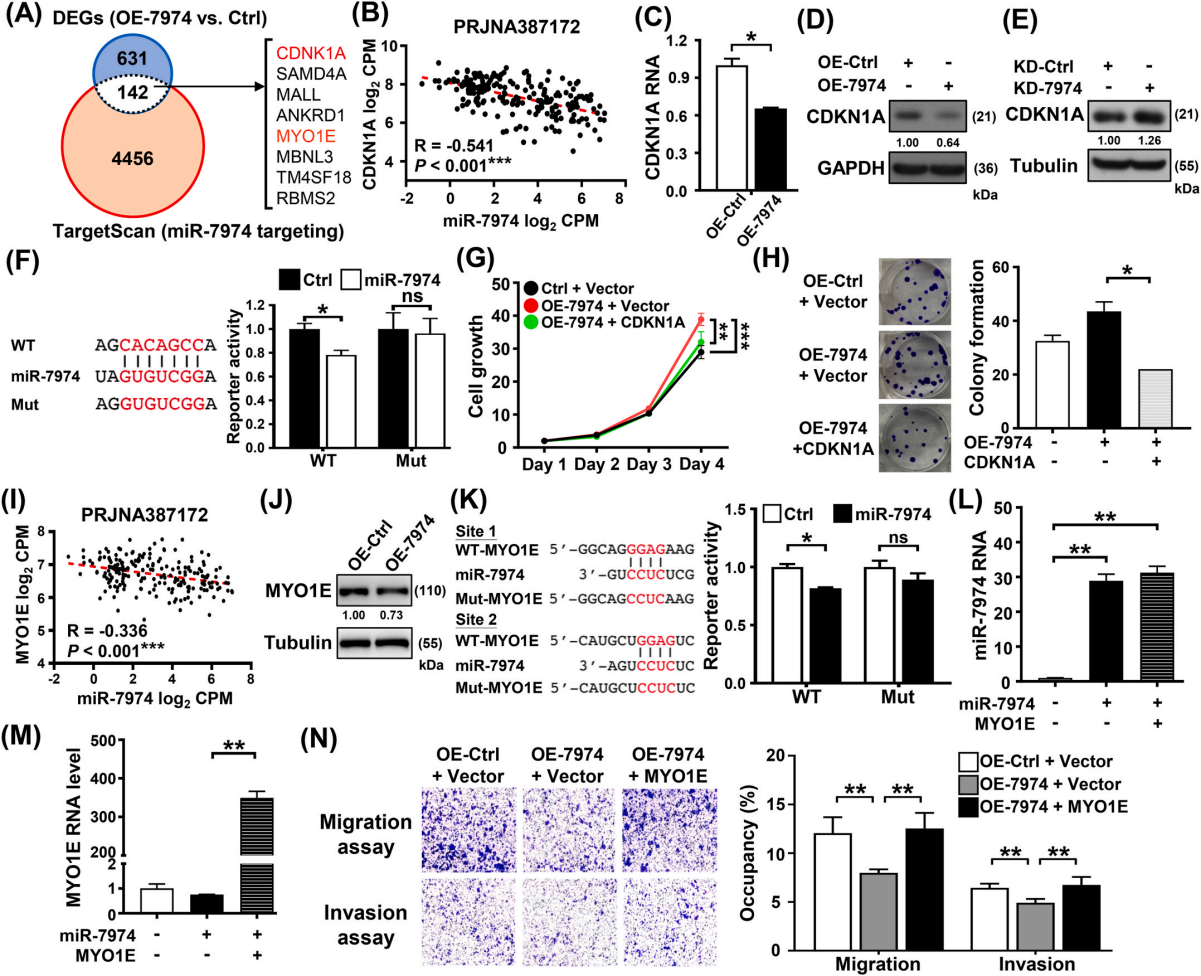
**MiR-7974 repression on CDKN1A and MYO1E promotes cancer cell growth and delays metastatic potential**. (A) The common genes by RNA-sequencing assays and TargetScan predictions were uncovered and showed in a Venn diagram. (B) MiRNA-7974 and CDKN1A reads from CRC-sequencing were subjected to correlation analysis, and the obtained correlation coefficient and *P*-value were noted. (C) CDKN1A RNA expression of the cells were assessed by RT-qPCR assays. (D and E) CDKN1A protein expression of the indicated transfectants were analyzed by Western blot assays. GAPDH and Tubulin expression served as loading control for immunoblots. (F) Schematic representation for CDKN1A gene reporter assay was depicted in left panel, and the luciferase activity of the transfectants was shown in right panel. (G) Time-course cell proliferation assays of the indicated cells was monitored by using MTT method. (H) Colony formation assays of specific transfection were conducted by crystal violet staining, and the representative images and quantification for forming colonies were shown. (I) Read counts of miR-7974 and MYO1E genes from CRC-sequencing were subjected to correlation analysis, and the received correlation coefficient and the *P*-value were noted. (J) MYO1E protein expression of the control and miR-7974 overexpressing cells were analyzed using Western blot assays. Tubulin expression was used as the internal control for blotting. (K) Schematic representation for MYO1E gene reporter assay was depicted in left panel, and the luciferase activity of the indicated transfectant was shown in right panel. (L and M) MiR-7974 and MYO1E expression of the specific transfected cells were assayed by RT-qPCR experiments. (N) Transwell assays of the transfectants were performed to evaluate cell metastasis ability, and the representative photos and quantification were shown.

To further identify direct targets of miR-7974, we intersected TargetScan-predicted 3′ UTR binding genes with the differentially expressed genes identified from miR-7974 overexpressing HCT-116 cells [Fig. 3E], yielding 142 candidate targets with both predicted binding sites and significant expression changes [Fig. 4A]. Among these, CDKN1A was notably downregulated in miR-7974-overexpressing cells and was implicated in the cyclin and cell cycle regulatory pathways highlighted by our RNA-seq data [Fig. S5]. Furthermore, co-expression analysis using matched mRNA and small RNA sequencing data from our cohort of 104 CRC patients (PRJNA387172) revealed a strong inverse correlation between CDKN1A and miR-7974 expression [Fig. 4B], suggesting that miR-7974 may promote cell cycle progression through suppression of CDKN1A. Experimental validation demonstrated that miR-7974 overexpression significantly reduced CDKN1A expression at both the mRNA and protein levels in HCT-116 cells [Fig. 4C and 4D], whereas LNA-mediated knockdown of miR-7974 resulted in increased CDKN1A protein expression [Fig. 4E]. To validate whether CDKN1A is a direct target of miR-7974, we performed a 3′ UTR luciferase reporter assay using constructs containing either the wild-type or a mutant form of the CDKN1A 3′ UTR ([Fig. 4F], left panel). Ectopic expression of miR-7974 significantly suppressed luciferase activity driven by the wild-type 3′ UTR, whereas mutation of the predicted miR-7974 binding site abrogated this repression ([Fig. 4F], right panel), thereby confirming a direct interaction between miR-7974 and the CDKN1A transcript.

Given the critical role of CDKN1A in regulating G1-S phase transition, we performed a rescue experiment to determine whether restoring CDKN1A expression could counteract the proliferative effects driven by miR-7974. An exogenous, epitope-tagged CDKN1A construct was introduced into HCT-116 cells stably overexpressing miR-7974. RT-qPCR analysis confirmed sustained miR-7974 overexpression following transfection [Fig. S6]. Functional assays revealed that enforced CDKN1A expression markedly reversed the enhanced proliferation and clonogenicity induced by miR-7974 overexpression [Fig. 4G and 4H]. These results support CDKN1A as a direct downstream effector functionally suppressed by miR-7974, mediating its oncogenic effects through modulation of cell cycle progression and proliferative capacity in CRC cells.

### 3.5 MiR-7974 attenuates cell metastasis by targeting MYO1E expression

Given the dual functional role of miR-7974 in promoting cell proliferation while suppressing metastasis, we next investigated whether additional downstream targets may contribute to its anti-metastatic effects in CRC. Using the same target identification strategy described above [Fig. 4A], we identified MYO1E, a member of the non-muscle class I myosins previously reported to promote cancer cell migration and invasion [27]. Consistently, MYO1E knockdown significantly impaired the migratory and invasive abilities of CRC cells [Fig. S7A], supporting its functional role in metastasis. Furthermore, an inverse correlation between MYO1E and miR-7974 expression was observed in our clinical CRC tissue cohort (PRJNA387172) [Fig. 4I], indicating a potential regulatory interaction. Western blot analysis revealed that MYO1E protein levels were markedly reduced in miR-7974 overexpressing cells [Fig. 4J]. To test whether MYO1E is a direct target of miR-7974, we performed a luciferase reporter assay using constructs containing either the wild-type or mutant 3′ UTR of MYO1E ([Fig. 4K], left panel). Overexpression of miR-7974 significantly repressed luciferase activity of the wild-type MYO1E reporter, whereas this effect was abolished in the mutant construct with disrupted miR-7974 binding sites ([Fig. 4K], right panel), confirming direct post-transcriptional targeting.

Given the pro-metastatic function of MYO1E [Fig. S7A], we next conducted a rescue experiment to determine whether restoring MYO1E expression could reverse the anti-metastatic phenotype induced by miR-7974. An epitope-tagged MYO1E expression construct was transfected into miR-7974-overexpressing HCT-116 cells, and RT-qPCR confirmed the co-expression of miR-7974 and MYO1E [Fig. 4L and 4M]. Transwell migration and invasion assays revealed that ectopic MYO1E expression restored the suppressed migratory and invasive capacity of miR-7974-overexpressing cells [Fig. 4N]. Although the effect of miR-7974 on invasion appeared moderate, repeated experiments consistently demonstrated this trend. Together, these results identify MYO1E as a direct and functional downstream effector of miR-7974 that mediates its anti-metastatic activity, complementing the previously established miR7974-CDKN1A axis in cell cycle regulation and proliferation. This highlights the context-dependent and dual regulatory roles of miR-7974 in CRC progression.

### 3.6 Downregulated miR-7974 expression in highly metastatic colorectal cancer cells

**Fig. 5:**
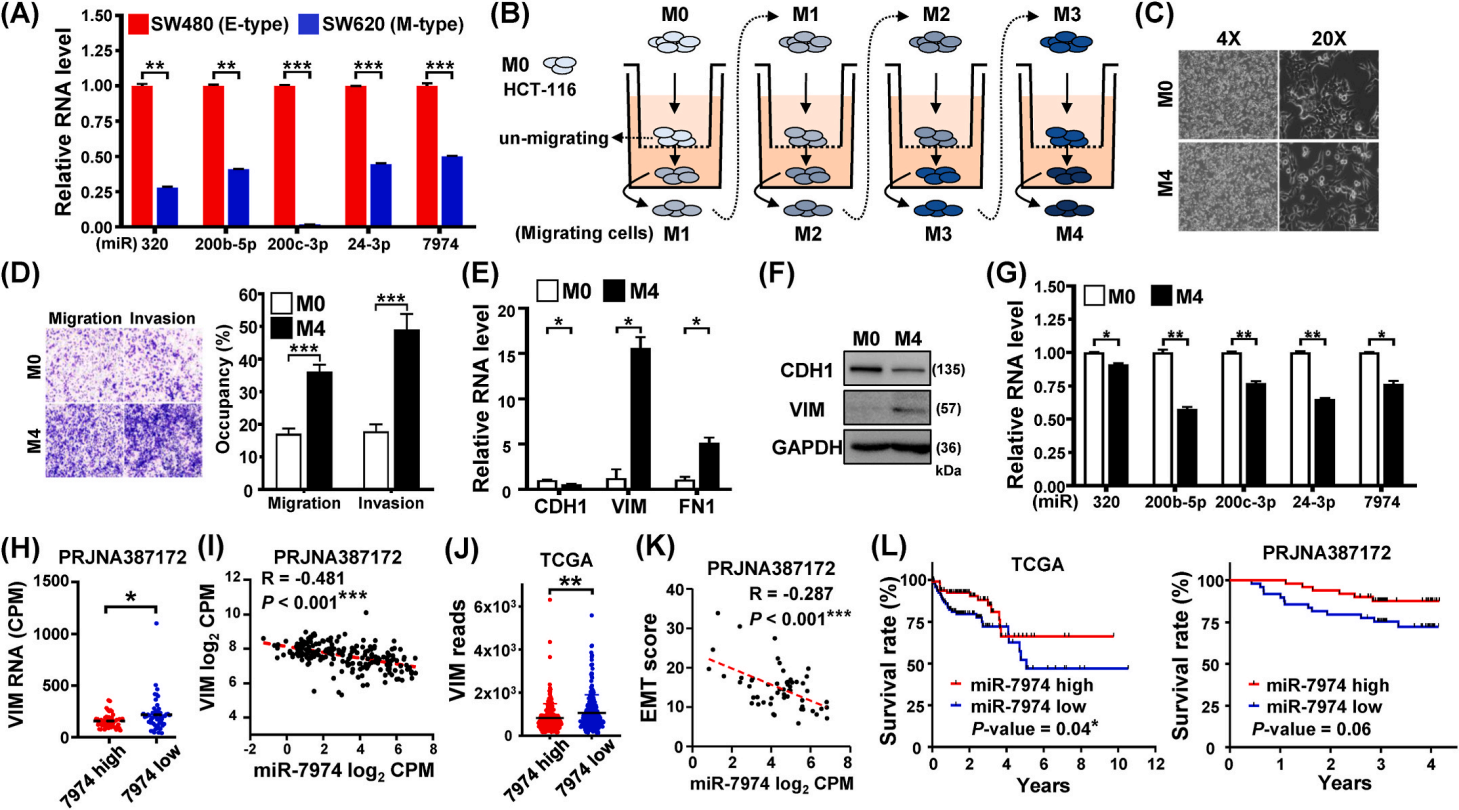
**MiR-7974 expression provides favorable regulation on cell growth but negatively regulates cell metastasis.** (A) Indicated miRNA expression of SW480 and SW620 cells were analyzed using RT-qPCR assays. (B) Schematic representation for the generation of highly migrating cells by Transwell-based approach was illustrated. (C) Cell morphology of M0 and M4 cells was recorded by microscopy. (D) Transwell experiments of the indicated cells were performed to evaluate cell metastasis ability. Representative photographs were shown in the left panel. (E) The indicated gene expression of cells was analyzed by RT-qPCR assays. (F) CDH1 and VIM protein expression in the M0 and M4 cells were monitored by Western blot assays. GAPDH expression was used as the loading control. (G) Target miRNA expression of cells was analyzed by RT-qPCR experiments. (H) VIM expression of the differentiated miR-7974 expressed samples from the CRC-sequencing were illustrated. (I) Read counts of miR-7974 and VIM genes from CRC-sequencing were illustrated in a dot graph and the correlation coefficient and P-value are noted in the lower left panel. (J) VIM gene expression of the differentiated miR-7974 expressed samples from TCGA resource were depicted. (K) MiR-7974 read counts and EMT scores of the CRC samples were subjected to correlation analysis, and the obtained correlation coefficient and P-value were noted. (L) Kaplan-Meier survival analyses of patients were performed in correspondence to miR-7974 expression from TCGA and CRC datasets. The statistical significance was noted in lower panels.

The contrasting effects of miR-7974 on proliferation and metastasis underscore its complex and context-dependent role in CRC progression. While miR-7974 is consistently overexpressed in tumor tissues compared to adjacent normal tissues, its expression is significantly higher in early-stage CRC and declines progressively in advanced stages [Fig. 1E]. This dynamic expression pattern suggests that elevated miR-7974 levels may support early tumor growth by promoting proliferation, whereas its downregulation may be required for the acquisition of metastatic traits. We therefore propose that, once tumor formation has occurred, sustained high expression of miR-7974 may provide a favorable outcome for cancer cells by restraining metastatic potential, thereby modulating CRC malignancy in a stage-specific manner. To validate our hypothesis in CRC progression, we examined its expression in a matched cell line pair derived from the same patient: SW480 cells, representing the primary tumor, and SW620 cells, isolated from a lymph node metastasis and characterized by enhanced metastatic potential. Consistent with our proposed model, miR-7974 expression was markedly lower in the metastatic SW620 cells compared to the more proliferative SW480 cells [Fig. 5A]. This expression pattern parallels that of other well-established metastasis suppressive microRNAs, such as the miR-200 family and miR-320 [9,[28], [29], [30]]. Conversely, MYO1E, a metastasis-promoting gene directly targeted by miR-7974-exhibited higher expression in SW620 than in SW480 cells, based on RNA sequencing data [Fig. S7B and S7C], further supporting the functional significance of the miR-7974-MYO1E regulatory axis in controlling metastatic potential.

To further investigate the dynamic role of miR-7974 in CRC malignancy, we employed a Transwell-based in vitro selection system to enrich for highly migratory HCT-116 subpopulations through four successive rounds of migration [Fig. 5B]. The resulting M4 subline exhibited markedly enhanced migratory morphology and metastatic potential compared to the original parental (M0) cells [Fig. 5C and 5D], validating the utility of this system for modeling progressive metastatic behavior. To characterize the EMT status of these subpopulations, we assessed the expression of EMT-associated markers. M0 cells exhibited higher levels of the epithelial marker CDH1 gene (E-cadherin), whereas M4 cells showed elevated expression of mesenchymal markers, including VIM (vimentin) and FN (fibronectin) genes, indicative of a more invasive phenotype [Fig. 5E and 5F]. In line with findings from the SW480 and SW620 cell line comparison, expression levels of miR-7974, as well as metastasis-suppressive miRNAs such as the miR-200 family and miR-320, were significantly reduced in the metastatic M4 cells compared to the more proliferative M0 population, further supporting their roles in regulating CRC progression [Fig. 5G].

### 3.7 Stage-dependent miR-7974 expression during colorectal cancer progression

Building on insights from our cell line experiments, we further examined miR-7974 expression in our clinical CRC tissue cohort (PRJNA387172) and in the TCGA dataset. In both datasets, tumors with high miR-7974 expression exhibited significantly lower levels of VIM [Fig. 5H to 5J)]. Correlation analyses revealed a strong inverse relationship between miR-7974 and VIM expression levels [Fig. 5I]. Conversely, MYO1E, the metastasis-promoting gene identified as a direct downstream target of miR-7974, showed a significant positive correlation with VIM expression [Fig. S7D], further supporting the involvement of the miR7974-MYO1E regulatory axis in EMT and metastatic progression. To comprehensively assess the clinical relevance of miR-7974 in EMT regulation, we constructed an EMT gene signature based on hallmark genes from the EMT pathway. An EMT score was computed for each patient sample, with higher scores indicating a more mesenchymal and potentially metastatic phenotype [26]. Correlation analysis between miR-7974 expression and EMT scores revealed a significant inverse relationship [Fig. 5K], reinforcing the hypothesis that miR-7974 suppresses EMT-associated transcriptional programs in CRC. Additionally, we applied consensus molecular subtype (CMS) classification to our CRC tissue cohort (PRJNA387172). Notably, miR-7974 expression was significantly higher in CMS2 tumors, a canonical subtype associated with active cell cycle and proliferation pathways, compared to CMS4 tumors, which are characterized by a mesenchymal and invasive phenotype [Fig. S8]. Interestingly, although miR-7974 was found to be consistently upregulated in tumor tissues compared to adjacent normal tissues, its expression progressively decreased across advancing CRC stages [Fig. 1]. To further explore the clinical relevance of this stage-dependent expression pattern, we analyzed overall survival data in the TCGA cohort. Notably, high miR-7974 expression was significantly associated with improved patient prognosis ([Fig. 5L], left panel; *p* < 0.05). A similar trend was observed in CRC cohort (PRJNA387172), although the association did not reach statistical significance ([Fig. 5L], right panel; *p* = 0.06).

**Fig. 6:**
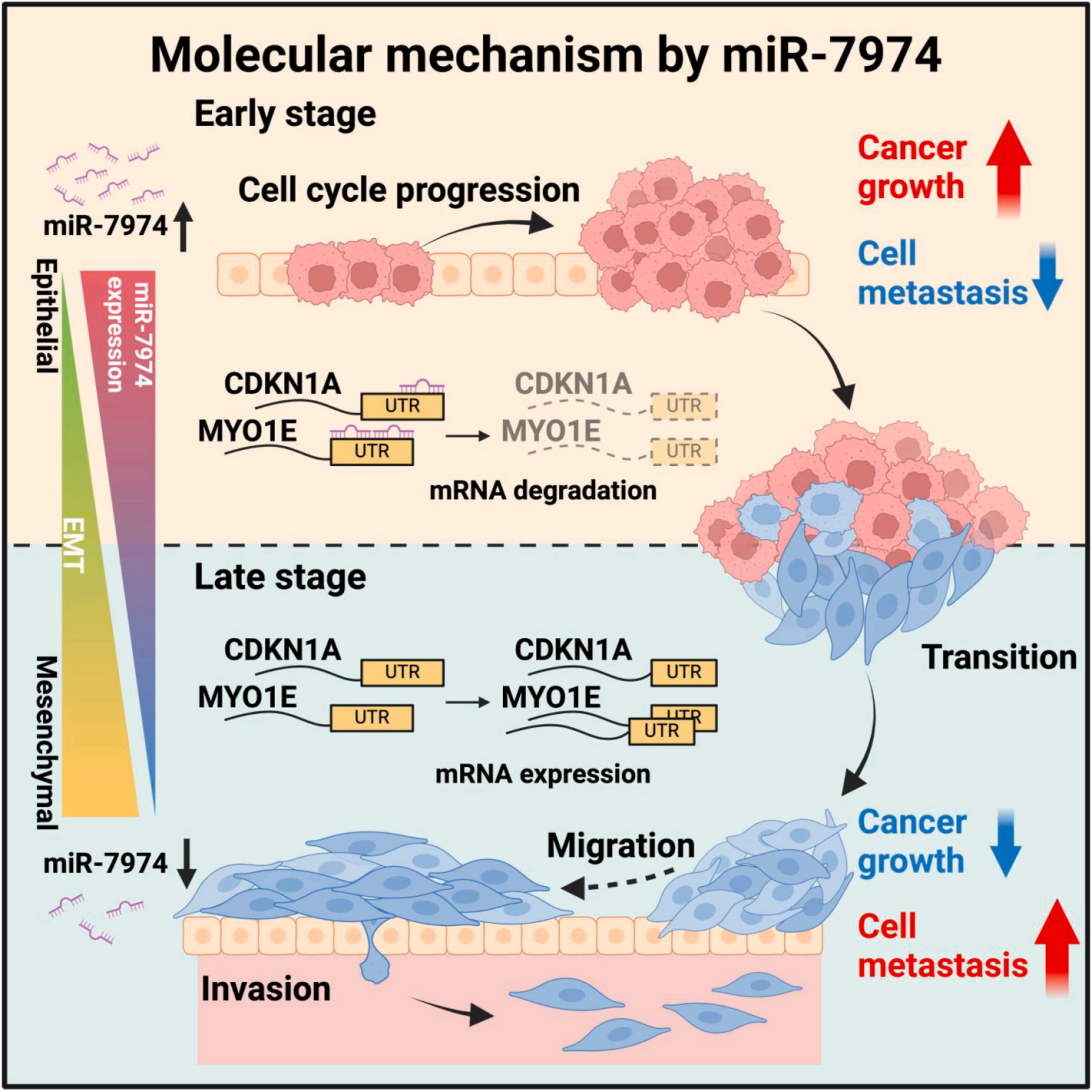
**Overview of the miR-7974 mediated molecular regulations during CRC progression.** Schematic illustration created with BioRender.com, is shown for miR-7974 mediated molecular regulation and its functional outcome for colorectal cancer development and progression.

Collectively, our findings demonstrate that miR-7974 exerts a stage-specific regulatory role in colorectal cancer progression [Fig. 6]. In early-stage tumors, elevated miR-7974 expression promotes proliferation by directly suppressing CDKN1A, whereas its downregulation in advanced stages facilitates invasion and metastasis via de-repression of MYO1E. This dynamic expression profile highlights miR-7974 as a modulator of EMT-MET plasticity, favoring a proliferative epithelial state during tumor initiation and enabling mesenchymal transition and dissemination as the disease progresses. These findings underscore the biological significance of miR-7974 in CRC progression and support its potential utility as a novel prognostic biomarker reflecting disease aggressiveness.

## 4 Discussion

MicroRNAs function as oncogenes or tumor suppressors, thereby influencing tumor progression [[31], [32], [33]]. Functional characterization of tumor-associated miRNAs provides important insights into cancer biology. Accordingly, we performed comprehensive transcriptomic profiling of 104 ethnic CRC patients. Systematic sequencing revealed a dysregulated miRNome in CRC development and progression. We subsequently identified a tumor-biased expression of one miRNA, miR-7974, which downregulated CDKN1A expression. This suppression accelerated cell cycle progression and consequently contributed to CRC cell growth, suggesting the diagnostic potential of miR-7974 in CRC. Furthermore, we discovered that miR-7974 negatively regulated cell metastasis by targeting MYO1E expression. Finally, we confirmed that stage-dependent downregulation of miR-7974 was associated with the acquisition of metastatic potential. These finding demonstrate the dual role of miR-7974 in CRC proliferation and metastasis, and further highlight the regulatory complexity of the miRNome in CRC progression.

Our current results reveal that the stage-dependent expression of miR-7974 closely correlates with the acquisition of metastatic potential in CRC cells, as demonstrated in both the SW480/SW620 cell model and in vitro developed migration cell lines (M0 and M4). Notably, the dynamic regulation of miR-7974 expression appears to facilitate a phenotypic switch from a proliferative to a metastatic state. This regulatory pattern is similar to that observed for the miR-200 family, particularly miR-200c, which has been extensively reported to preserve the epithelial phenotype by directly targeting the EMT-inducing transcription factors ZEB1 and ZEB2 [24,34,35]. Through this mechanism, miR-200c modulates EMT plasticity and promotes MET during metastatic colonization. Epigenetic regulation, particularly DNA methylation, has been shown to control miR-200c expression in metastatic CRC, thereby suppressing EMT and facilitating re-epithelialization at distant sites [24]. Given that miR-7974 also exhibits stage-specific expression patterns—particularly reduced levels in advanced metastatic stages—this raises the possibility that similar epigenetic mechanisms may contribute to its downregulation. Further investigation is warranted to determine whether epigenetic regulation of miR-7974 in late-stage CRC influences its potential role in modulating EMT–MET plasticity.

Despite our novel findings, one of the primary limitations of this study lies in the use of bulk RNA sequencing, which, while informative, is constrained by sample heterogeneity both within and between patient samples. Bulk RNA sequencing captures an aggregate signal from all cell types in the tumor microenvironment, which includes not only tumor cells but also immune and stromal cells. This can obscure the specific expression patterns of miR-7974 within tumor cells, as signals from surrounding immune and stromal cells may confound our interpretation of miR-7974's role in tumor EMT processes. To more accurately investigate miR-7974's function in EMT dynamics within tumor cells specifically, single-cell RNA sequencing would provide a more precise approach, allowing us to isolate miR-7974 expression at the cellular level and track its regulation across distinct cell populations within the tumor. This method could clarify miR-7974's specific contributions to EMT progression and provide deeper insights into its role in tumor cell plasticity.

We recognized a notable difference in miR-7974 expression (i.e., read count) between the two data sources using our in-house database and TCGA resources. Therefore, we propose its attribution to the discrepancy of the microbiota spectrum. Accumulating evidence supports the bidirectional association between the miRNome and microbiota. In particular, *Fusobacterium nucleatum* has upregulated miRNA-21 expression to promote CRC growth [36] and fine-tuned the autophagy mechanism, thereby increasing CRC chemoresistance [37]. Conversely, the miRNome regulated the microbiota, such as 16S/23S rRNA of *F. nucleatum* targeting by miRNA-515-5p or VegH gene modification by miRNA-1226-5p in *Escherichia coli* [38]. Hence, the negative correlation between miR7974 and *Blautia* may indicate a particular interaction with regional specificity that elucidates the crosstalk of miRNome and microbiota [39]. Conversely, the miR-7974 gene is located on chromosome 19q in the intron region of the ZNF653 gene. However, ZNF653 gene expression remains stable, contrary to the varying miR-7974 gene expression. This excludes the potential for putative host gene activation/deactivation for the dynamic miR-7974 expression. A rational explanation for the variable expression of miRNA-7974 is attributed to the dysregulated miRNA processing during cancer development [40]. However, advanced validation warrants additional supportive findings.

## Data availability statement

The RNA-sequencing data of HCT-116 cells stably overexpressing miR-7974, along with control cells, have been deposited in the NCBI Gene Expression Omnibus (GEO) database under the accession code GSE214255. The original blots were included in the last figure of supplemental information.

## Ethics statement

The animal experiments were approved by Laboratory Animal Center, Chang Gung University (CGU111-121).

## Author contributions

WST and HL contributed to the conception of the study. YHL, YTC, YCC, CPM, PTH, and YHL performed the experiments. YHL, YTC, YCC, EN, YYL, IYFC, CYY, and JSY analyzed the data. WST, HCH, and HL supervised the study. YTC, EN, YYL, YCC, IYFC, BCMT, CYY, and HL wrote the manuscript; BCMT, CYY, JSY, HCH, and HL revised the manuscript. All authors read and approved the final manuscript.

## Clinical trial number

Not Applicable.

## Declaration of generative AI and AI-assisted technologies in the writing process

During the preparation of this work the author(s) used ChatGPT in order to do grammatical editing. After using this tool/service, the author(s) reviewed and edited the content as needed and take(s) full responsibility for the content of the publication.

## Funding information

This research was supported by grants from the National Science and Technology Council, Taiwan (NSTC 110-2320-B-182-015-MY3 and NSTC 113-2320-B-182-016-MY3 to HL) and the Chang Gung Memorial Hospital (CMRPD1M0901-2 and BMRPF45 to HL).

## Declaration of competing interest

The authors declare that they have no known competing financial interests or personal relationships that could have appeared to influence the work reported in this paper.
