# Supplementary material for: Functional dynamic modulation of colorectal cancer initiation and metastatic capacity by a novel MiR-7974 regulatory axis
Source: Biomed J. 2025 Nov 28;49(4):100934. doi: 10.1016/j.bj.2025.100934 (PMC13453470; doi:10.1016/j.bj.2025.100934)

Functional Dynamic Modulation of Colorectal Cancer Initiation and Metastatic Capacity by a Novel MiR-7974 Regulatory Axis

Yu-Hao Liu[1,#], Yi-Tung Chen[2,3,#], Yu-Chang Chen[4], En Chin[4], Ying-Yu Lai[4], Chung-Pei Ma[3], Ian Yi-Feng Chang[2,5], Bertrand Chin-Ming Tan[3,4,5,6], Pei-Ting Hsu[4], Yi-Hsuan Lai[3], Wen-Sy Tsai[7], Chia-Yu Yang[2,4,8,9], Jau-Song Yu[1,2,4,10], Hung-Chih Hsu[11,12], Hsuan Liu[1,2,4,11,*]

[1]Department of Cell and Molecular Biology, College of Medicine, Chang Gung University, Taoyuan, Taiwan
[2]Molecular Medicine Research Center, Chang Gung University, Taoyuan, Taiwan
[3]Department of Biomedical Sciences, College of Medicine, Chang Gung University, Taoyuan, Taiwan
[4]Graduate Institute of Biomedical Sciences, College of Medicine, Chang Gung University, Taoyuan, Taiwan
[5]Department of Neurosurgery, Lin-Kou Medical Center, Chang Gung Memorial Hospital, Taoyuan, Taiwan
[6]Research Center for Emerging Viral Infections, Chang Gung University, Taoyuan, Taiwan
[7]Division of Colon and Rectal Surgery, Lin-Kou Medical Center, Chang Gung Memorial Hospital, Taoyuan, Taiwan
[8]Department of Microbiology and Immunology, College of Medicine, Chang Gung University, Taoyuan, Taiwan
[9]Department of Otolaryngology-Head & Neck Surgery, Chang Gung Memorial Hospital, Linkou, Taoyuan, Taiwan
[10]Liver Research Center, Chang Gung Memorial Hospital at Linkou, Taoyuan, Taiwan.
[11]Division of Hematology-Oncology, Department of Internal Medicine, Chang Gung Memorial Hospital at Linkou, Taoyuan, Taiwan.
[12]College of Medicine, Chang Gung University, Taoyuan, Taiwan

# These authors contributed equally to this paper
* Correspondence: liu-hsuan@mail.cgu.edu.tw

Supplemental Information

Table S1. List of primers used in this study.

**Primers**	**Sequence (5' to 3')**	**Application**
miR-RNU6-Forward	CAAATTCGTGAAGCGTTCCA	RT-qPCR
Universal-Reverse	CAACTGGTGTCGTGGAGTCGG	
miR-7974-Forward	CGGCGGAGGCTGTGATGCTCTC	RT-qPCR
Universal-Reverse	CAACTGGTGTCGTGGAGTCGG	
miR-24-3p-Forward	CGGCGGTGGCTCAGTTCAGCAG	RT-qPCR
Universal-Reverse	CAACTGGTGTCGTGGAGTCGG	
miR-200c-3p-Forward	CGGCGGTAATACTGCCGGGTAATG	RT-qPCR
Universal-Reverse	CAACTGGTGTCGTGGAGTCGG	
miR-200c-5p-Forward	CGGCGGCATCTTACTGGGCAGC	RT-qPCR
Universal-Reverse	CAACTGGTGTCGTGGAGTCGG	
miR-320c-Forward	CGGCGGAAAAGCTGGGTTGAGAG	RT-qPCR
Universal-Reverse	CAACTGGTGTCGTGGAGTCGG	
EEF1A1-Forward	CAATGTGGGCTTCAATGTCAA	RT-qPCR
EEF1A1-Reverse	CATAGCCGGCGCTTATTTG	
CDNK1A-Forward	CCAGCATGACAGATTTCTACCA	RT-qPCR
CDNK1A-Reverse	AAGATGTAGAGCGGGCCTTT	
RBMS2-Forward	TGCAGACATGGGCGTCAT	RT-qPCR
RBMS2-Reverse	GCAGACTGAGCAAGCATCCTG	
MYO1E-Forward	CAAGGAGCGGCACAGTATG	RT-qPCR
MYO1E-Reverse	CACTGTTTTTCCAGCACCAC	
CDH1-Forward	CAAGGAGCGGCACAGTATG	RT-qPCR
CDH1-Reverse	ACCCTTGTACGTGGTGGG	
VIM-Forward	CAGACAGGATGTTGACAATGC	RT-qPCR
VIM-Reverse	GGATTTCCTCTTCGTGGAGTT	
FN1-Forward	ACAAACAACAGCGCCTGAT	RT-qPCR
FN1-Reverse	ATTCTGTACCCTGTGATGGGA	
RT-RNU6	CTCAACTGGTGTCGTGGAGTCGGCAATTCAGTTGAGAAAATATGGAACG	Specific RT-primer

RT-miR-7974	CTCAACTGGTGTCGTGGAGTCGGCA ATTCAGTTGAGGGGCTCAGGA	Specific RT-primer
RT-miR-24-3p	CTCAACTGGTGTCGTGGAGTCGGCA ATTCAGTTGAGCTGTTCCTGC	Specific RT-primer
RT-miR-200c-3p	CTCAACTGGTGTCGTGGAGTCGGCA ATTCAGTTGAGTCCATCATTAC	Specific RT-primer
RT-miR-200c-5p	CTCAACTGGTGTCGTGGAGTCGGCA ATTCAGTTGAGTCCAATGCTG	Specific RT-primer
RT-miR-320c	CTCAACTGGTGTCGTGGAGTCGGCA ATTCAGTTGAGACCCTCTCAAC	Specific RT-primer
OE-miR-7974-Forward	TGCTGAGGCTGTGATGCTCTCCTGA GCCCGTTTTGGCCACTGACTGACGG GCTCAGGAGCATCACAGCCT	Construction
OE-miR-7974-Reverse	CCTGAGGCTGTGATGCTCCTGAGCC CGTCAGTCAGTGGCCAAAACGGGCT CAGGAGAGCATCACAGCCTC	
CDKN1A-3UTR-Forward	ACGTACTAGTCCTCAAAGGCCCGCT CTACA	Construction
CDKN1A-3UTR-Reverse	ACGTACGCGTGGATGAGGAAGGTCG CTGGA	
MYO1E-3UTR-Forward	GAGCTCCAGGCACAGACCAGGGGA GGGGAT	Construction
MYO1E-3UTR-Reverse	ACGCGTGGCCCTTTTGCATGTCTTTA AGCC	
MYO1E-3UTR-Mutant 1 -Forward	GTGAAAAGGCAGCCTCTAGTGTCGG ATTCACAAGCCC	Construction
MYO1E-3UTR-Mutant 1 -Reverse	GGGCTTGTGAATCCGACACTAGAGG CTGCCTTTTCAC	
MYO1E-3UTR-Mutant 2 -Forward	GAGGAGCATGCTCCTCTCCCGTGTC GGACCAGCACTCAG	Construction
MYO1E-3UTR-Mutant 2 -Reverse	AGTGCTGGTCCGACACGGGAGAGG AGCATGCTCCTCTGTCCCAG	
LNA-Ctrl	CAGUACUUUUGUGUAGUACA	RNAi
LNA-miR-7974	CUCAGGAGAGCAUCACAGCC	

Supplemental Figures & Legends

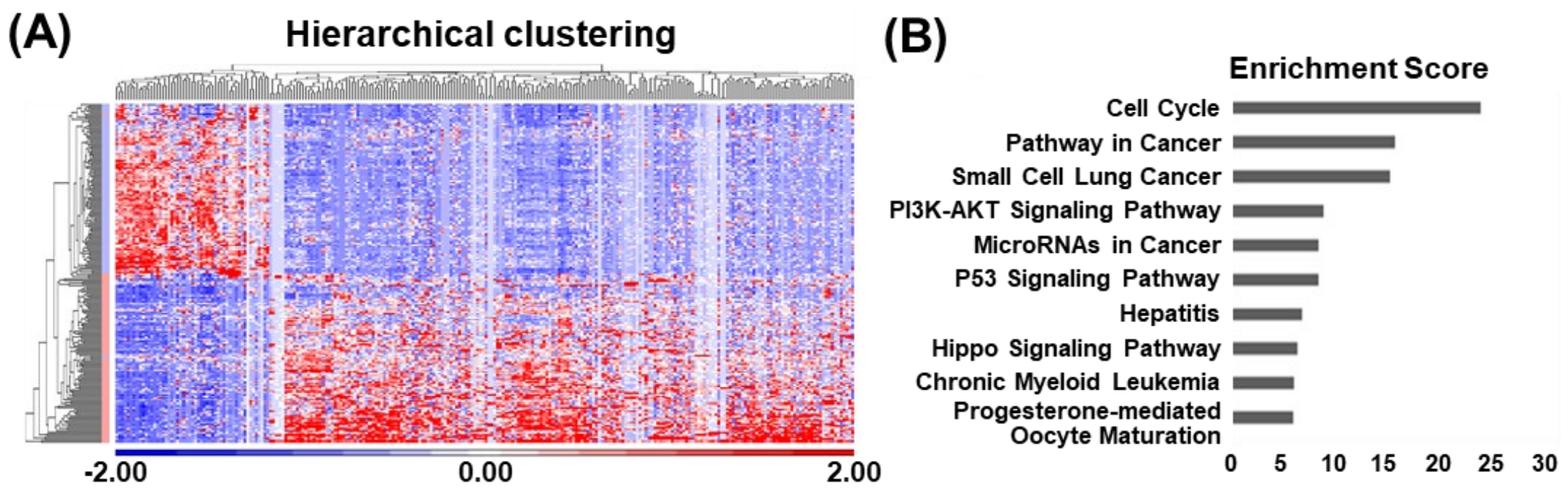

Figure S1. Differentiated expressed miRNAs between normal and CRC tumor tissues. Previous RNA sequencing data (PRJNA387172) were utilized to identify differentially expressed miRNAs between normal and colorectal cancer (CRC) tumor tissues. Applying filtering criteria of fold change ($|FC| \geq 1.5$) and statistical significance ($P < 0.05$), a total of 254 differentially expressed miRNAs were identified. Their expression patterns were visualized through hierarchical clustering. (B) These miRNAs were subsequently subjected to Gene Set Enrichment Analysis to uncover the enriched signaling pathways.

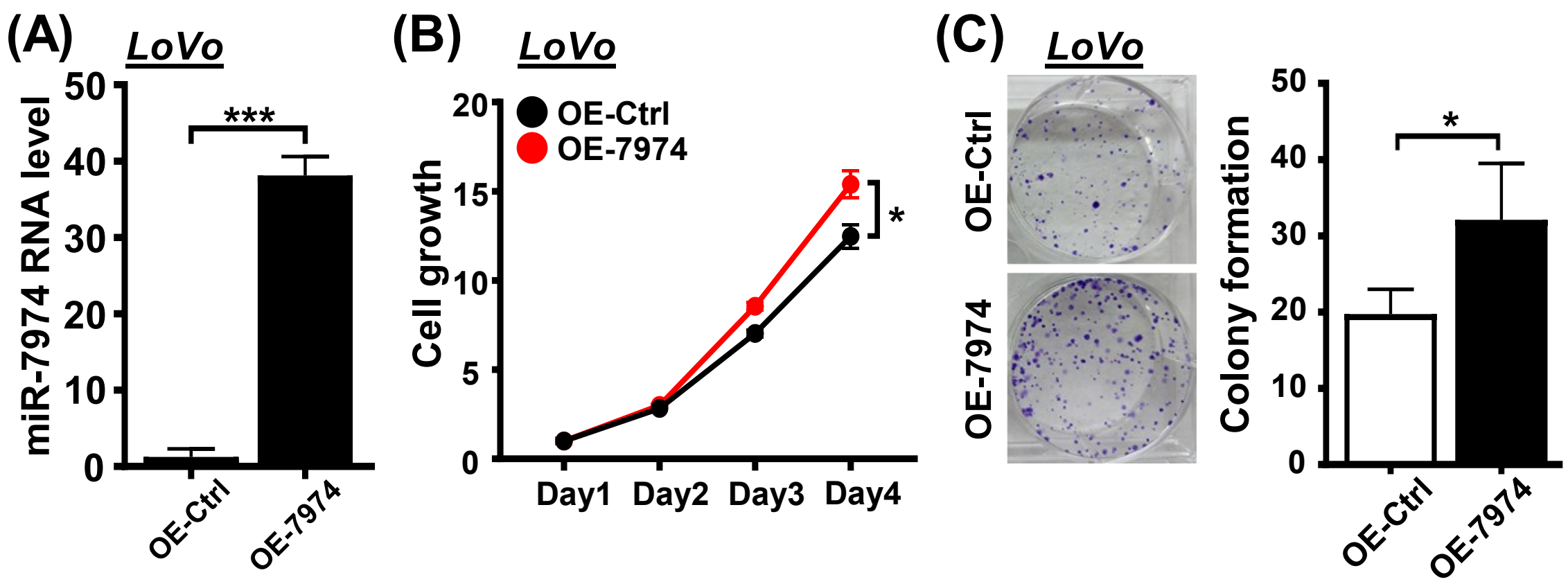

Figure S2. MiR-7974 overexpression enhances the LoVo cell proliferation and clonogenicity. (A) The miR-7974 expression of control and miR-7974 overexpressing (OE-7974) LoVo cells was analyzed by RT-qPCR assays (A), and the cell proliferation (B) and colony formation ability (C) of the cells was assessed by MTT assay and crystal violet staining respectively.

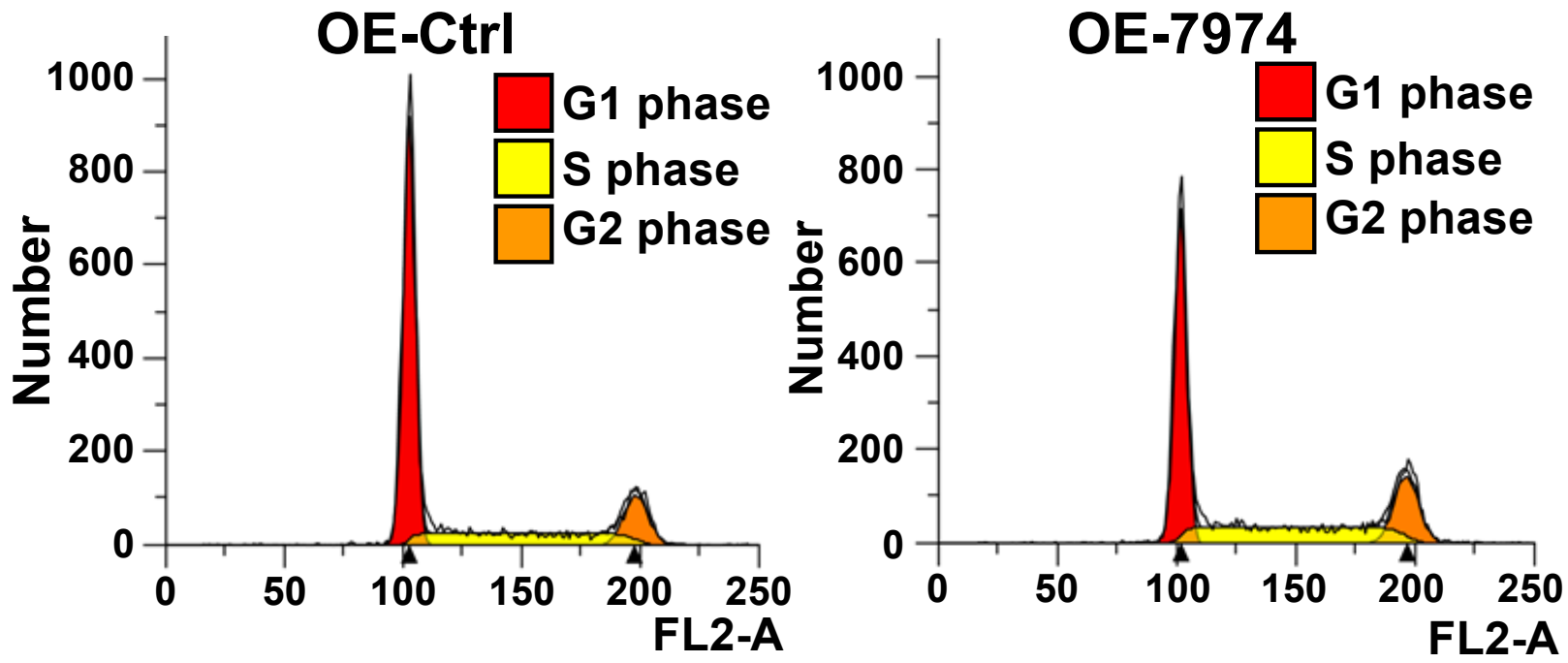

Figure S3. Overexpression of miR-7974 enhances cell cycle progression. Flow cytometry was performed on control and miR-7974 overexpressing (OE-7974) cells to evaluate cell cycle progression. The resulting cell cycle profiles are shown as histograms.

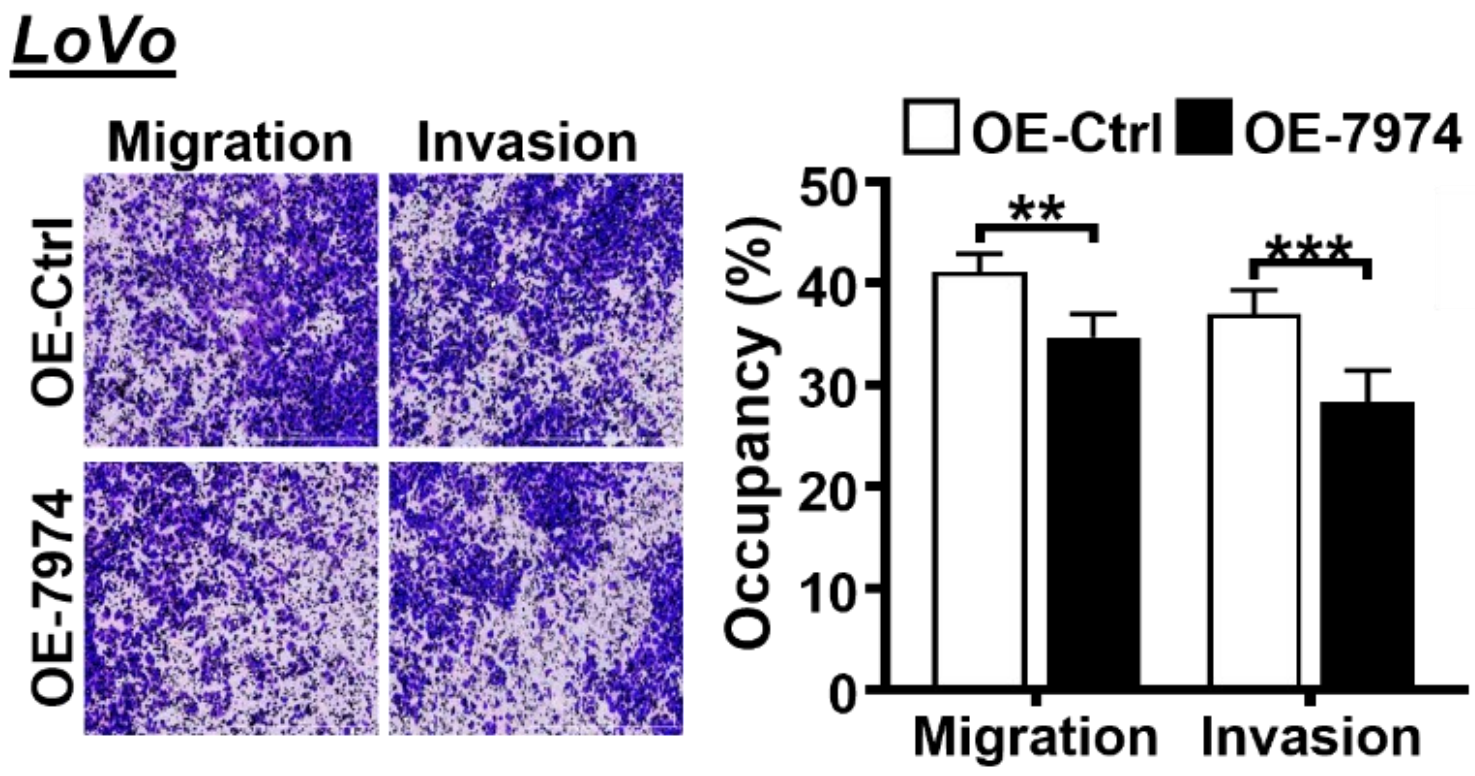

Figure S4. MYO1E knockdown downregulates cell metastatic potential. MYO1E knockdown cells in LoVo cells were subjected to Transwell migration and invasion assays, and the representative images and quantification for assays were shown.

No.	Ingenuity Canonical Pathways	-log(P)	Z-score	Molecules
1	Tumor Microenvironment Pathway	4.9	-2.84	FAS,FGF1,FGF8,FN1,IL6R,MMP13,MMP19,MMP24,MMP7,PDGFB,PDGFC,SLC2A1,SLC2A3,SPP1,TGFB2
2	HIF1-alpha Signaling	4.13	-3.87	ADRA1B,CDKN1A,EDN1,IL6R,MMP13,MMP19,MMP24,MMP7,PDGFB,PDGFC,SERPINE1,SLC2A1,SLC2A3,TGFB2,VIM
4	Osteoarthritis Pathway	3.79	-1.73	ALPP,CREB3L3,CREB5,FGF8,FN1,GLI2,H19,IL1RAP,ITGB5,MMP13,PDGFC,PRKAA2,RUNX2,SMAD7,SPP1
5	HOTAIR Regulatory Pathway	3.52	-1.73	ATXN1,CDH1,CDKN1A,MMP13,MMP19,MMP24,MMP7,SNAI2,SPP1,VIM,WNT7A,WNT9A
6	Synaptogenesis Signaling Pathway	3.51	1.50	BDNF,CACNB4,CDH1,CHN1,CNTNAP2,CREB3L3,CREB5,EFNA5,GRIN1,ITPR1,MAP1B,MAPT,MARCKS,RAB3A,SYN1,SYT11,SYT12,THBS1
7	p53 Signaling	3.41	-2.12	CDKN1A,FAS,GADD45G,HDAC9,PMAIP1,SERPINB5,SNAI2,THBS1,TP53I3
8	Hepatic Fibrosis Signaling Pathway	3.35	-2.52	CCN2,COL5A3,CREB3L3,CREB5,EDN1,EDNRA,GLI2,IL18,IL1RAP,IRS2,ITGB5,MMP13,PDGFB,PDGFC,SERPINE1,SMAD7,SPP1,TGFB2,WNT7A,WNT9A
9	GP6 Signaling Pathway	3.24	-2.53	COL12A1,COL24A1,COL28A1,COL4A2,COL5A3,COL6A3,ITPR1,LAMA3,LAMB3,LAMC2
10	AMPK Signaling	2.91	-0.63	ADRA1B,ADRB2,AK4,AK5,CDKN1A,CHRNB1,CREB3L3,CREB5,IRS2,PPM1D,PRKAA2,RAB3A,SLC2A1
11	Inhibition of Matrix Metalloproteases	2.74	1.34	MMP13,MMP19,MMP24,MMP7,TIMP2
13	ILK Signaling	2.37	-1.90	CDH1,CREB3L3,CREB5,FLNC,FN1,IRS2,ITGB5,ITGB8,PDGFC,SNAI2,VIM
14	Breast Cancer Regulation by Stathmin1	2.34	-0.63	ADRA1B,ADRB2,ARHGEF16,CDK6,CREB3L3,CREB5,CRHR1,EDNRA,F2RL2,FPR1,GNG4,GPR180,GPR183,GPR87,HTR1B,HTR7,MC1R,OPRD1,PDGFB,PDGFC,PLCB4,PTAFR,SSTR5,TGFB2
15	p38 MAPK Signaling	2.26	-1.63	CREB3L3,CREB5,FAS,IL18,IL1RAP,MAPT,RPS6KA1,TGFB2
17	VDR/RXR Activation	2.06	-1.00	CDKN1A,IGFBP6,NCOR2,RUNX2,SPP1,TGFB2
18	Cyclins and Cell Cycle Regulation	1.99	1.34	CCNB2,CDK6,CDKN1A,CDKN2B,HDAC9,TGFB2
19	Regulation Of The Epithelial Mesenchymal Transition By Growth Factors Pathway	1.96	-1.41	CDH1,FGF1,FGF8,IL6R,PDGFB,PDGFC,SNAI2,TGFB2,TNFSF12,VIM
20	MSP-RON Signaling In Cancer Cells Pathway	1.94	-2.12	CREB3L3,CREB5,ELF1,ELK3,KLK10,KLK5,PDGFC,VIM
21	Role of Hypercytokinemia/hyperchemokinemia in the Pathogenesis of Influenza	1.87	-1.63	DDX58,IFIT2,IFIT3,IL18,IRF7,OAS2
22	Phospholipase C Signaling	1.72	-0.71	ARHGEF16,CREB3L3,CREB5,GNG4,HDAC9,ITGB5,ITPR1,MARCKS,NFATC2,PLCB4,PLD1,TGM2
24	Cell Cycle: G2/M DNA Damage Checkpoint Regulation	1.6	-1.00	CCNB2,CDKN1A,PPM1D,RPS6KA1
25	Ephrin Receptor Signaling	1.54	1.00	CREB3L3,CREB5,EFNA5,FGF1,GNG4,GRIN1,ITGB5,PDGFB,PDGFC
26	Colorectal Cancer Metastasis Signaling	1.52	-2.33	CDH1,GNG4,IL6R,MMP13,MMP19,MMP24,MMP7,PDGFC,TGFB2,WNT7A,WNT9A
27	PPAR Signaling	1.49	0.82	CITED2,IL18,IL1RAP,NCOR2,PDGFB,PDGFC
28	Glioblastoma Multiforme Signaling	1.45	-1.13	CDK6,CDKN1A,ITPR1,PDGFB,PDGFC,PLCB4,WNT7A,WNT9A

Figure S5. Pathway annotation of the differentially expressed genes at miR-7974 overexpression. Pathway annotations of the differentially expressed genes by Ingenuity Pathway Analysis bioinformatics were performed and illustrated.

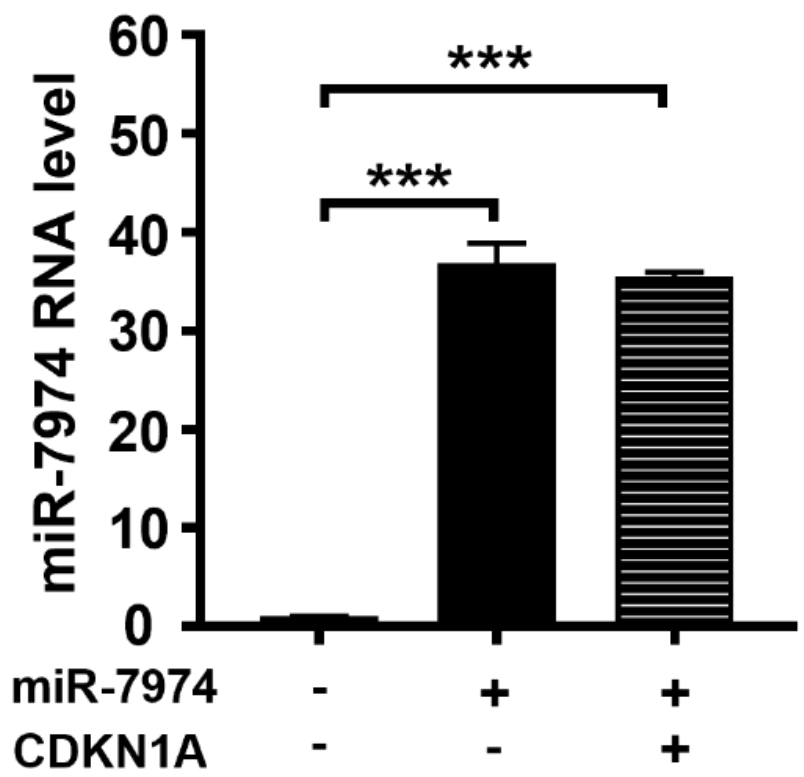

Figure S6. Ectopic CDKN1A expression in the miR-7974 overexpressing cells. MiR-7974 expression of the specific transfectants in **Figure 4G** was analyzed by RT-qPCR assays.

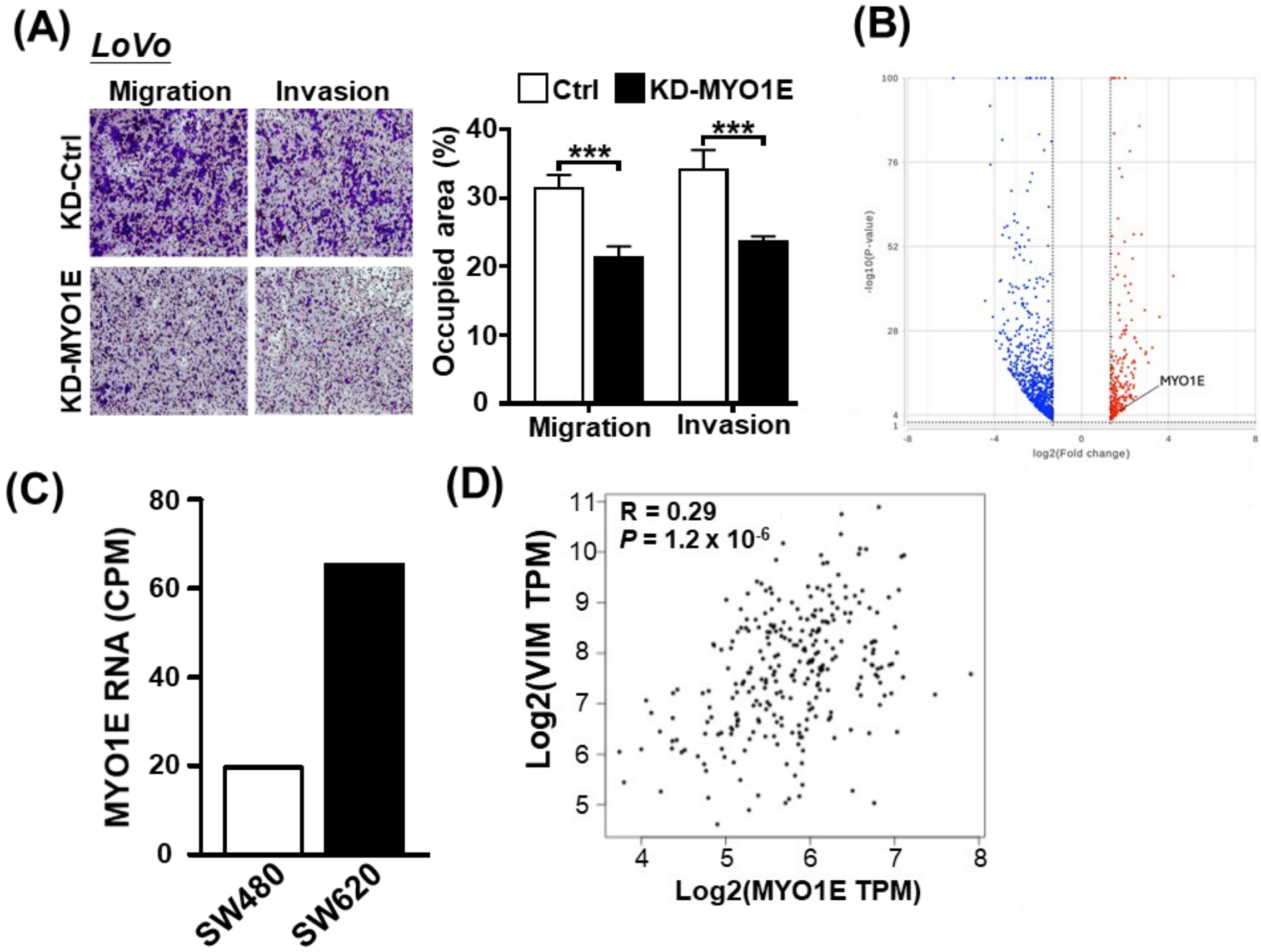

Figure S7. Knockdown of MYO1E expression downregulates cell metastatic potential. (A) Control and MYO1E knockdown LoVo cells were subjected to Transwell migration and invasion assays, and the representative images and quantification for assays were shown. (B) Transcriptomic profiles of SW480 and SW620 cells were performed, and the differentiated expressed genes between cells were depicted as volcano plot. (C) The MYO1E RNA expression level of cells was presented in a bar graph. (D) Correlation analysis of MYO1E and VIM genes was completed by public GEPIA2 database, and the received correlation coefficient and the *P*-value were noted.

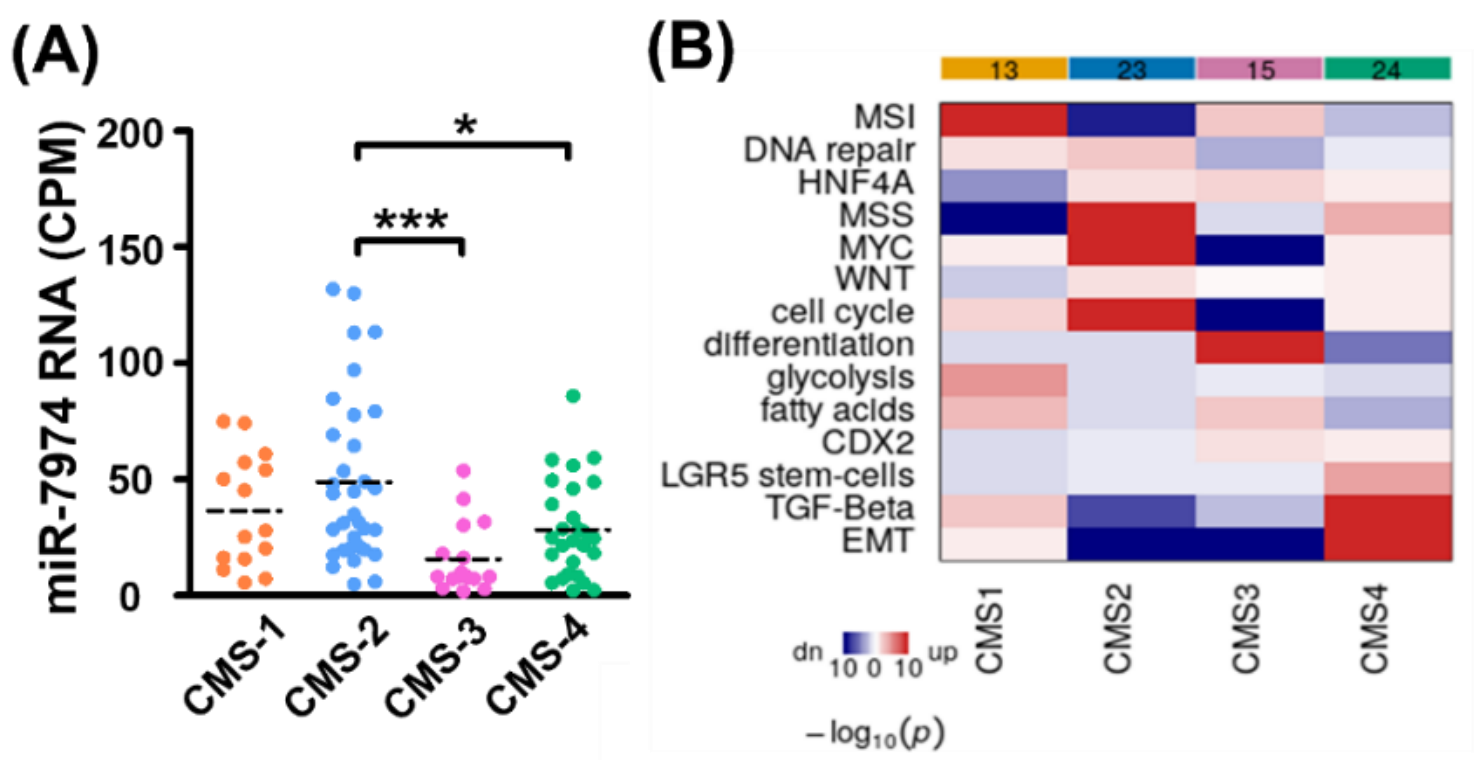

Figure S8. MiR-7974 RNA expression levels across CRC molecular subtypes. (A) Consensus Molecular Subtype (CMS) classification was applied to stratify CRC patients into molecular subtypes. The expression

levels of miR-7974 across the indicated CMS subtypes are presented. (B) Differentially expressed genes between CMS clusters were subjected to pathway enrichment analysis. The resulting expression patterns of representative pathways across CMS1 to CMS4 are visualized using a heatmap.

Original Western Blots

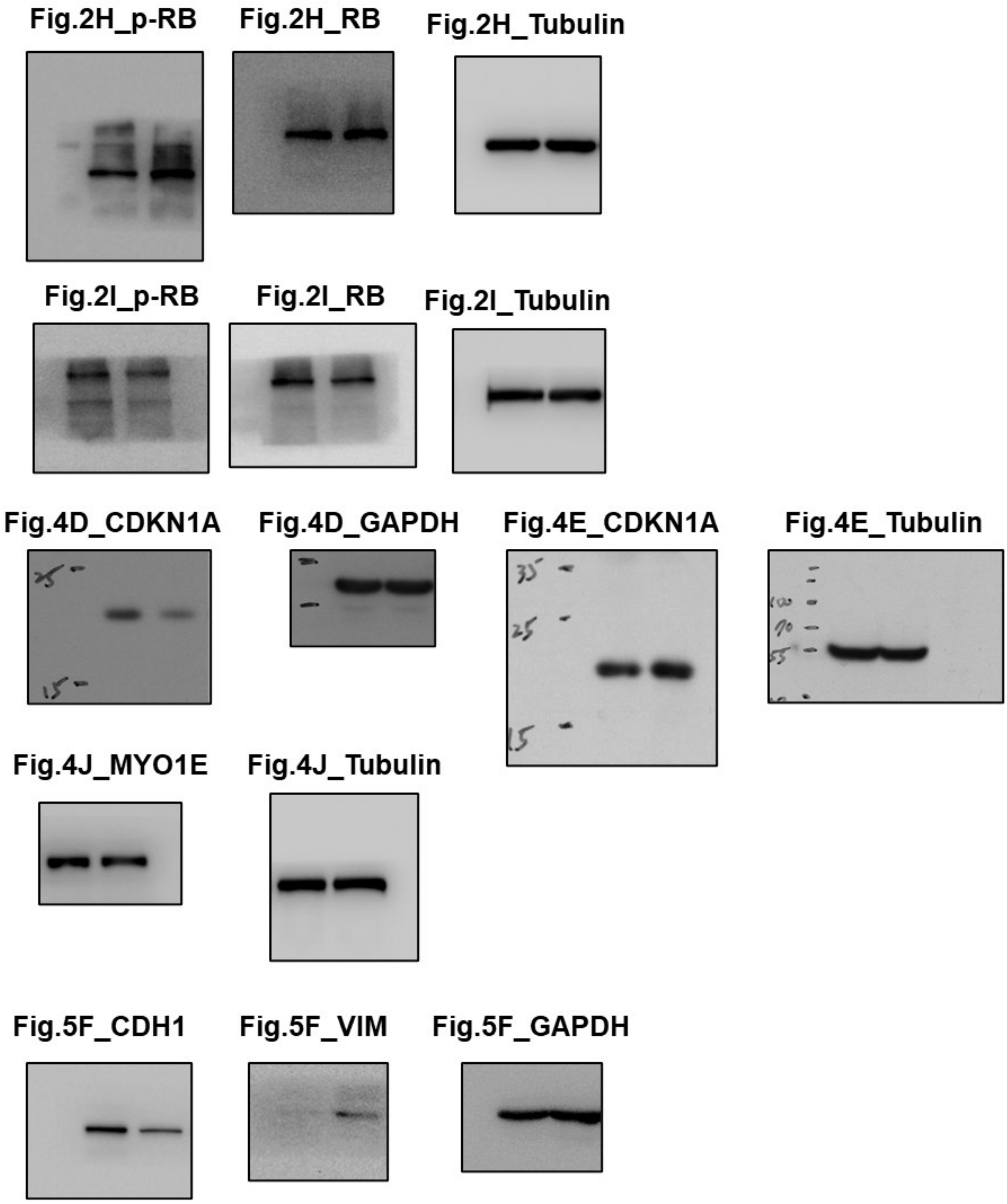

Supplement: Supplementary file 1 [file mmc1.pdf]
